# Gall volatiles defend aphids against a browsing mammal

**DOI:** 10.1186/1471-2148-13-193

**Published:** 2013-09-11

**Authors:** Michael Rostás, Daniel Maag, Makihiko Ikegami, Moshe Inbar

**Affiliations:** 1 Bio-Protection Research Centre, Lincoln University, PO Box 85084, Lincoln 7647, New Zealand; 2 Department of Botany II, University of Würzburg, Würzburg 97082, Germany; 3 Department of Evolutionary & Environmental Biology, University of Haifa, Haifa 31905, Israel; 4 Laboratory of Fundamental and Applied Research in Chemical Ecology, Institute of Biology, University of Neuchâtel, 2009, Neuchâtel, Switzerland

**Keywords:** *Capra hircus*, Enemy hypothesis, Extended phenotype, Herbivory, Intraguild predation, Plant defence, Tannins, Terpenes, Volatile organic compounds

## Abstract

**Background:**

Plants have evolved an astonishing array of survival strategies. To defend against insects, for example, damaged plants emit volatile organic compounds that attract the herbivore’s natural enemies. So far, plant volatile responses have been studied extensively in conjunction with leaf chewing and sap sucking insects, yet little is known about the relationship between plant volatiles and gall-inducers, the most sophisticated herbivores. Here we describe a new role for volatiles as gall-insects were found to benefit from this plant defence.

**Results:**

Chemical analyses of galls triggered by the gregarious aphid *Slavum wertheimae* on wild pistachio trees showed that these structures contained and emitted considerably higher quantities of plant terpenes than neighbouring leaves and fruits. Behavioural assays using goats as a generalist herbivore confirmed that the accumulated terpenes acted as olfactory signals and feeding deterrents, thus enabling the gall-inducers to escape from inadvertent predation by mammals.

**Conclusions:**

Increased emission of plant volatiles in response to insect activity is commonly looked upon as a “cry for help” by the plant to attract the insect’s natural enemies. In contrast, we show that such volatiles can serve as a first line of insect defences that extends the ‘extended phenotype’ represented by galls, beyond physical boundaries. Our data support the Enemy hypothesis insofar that high levels of gall secondary metabolites confer protection against natural enemies.

## Background

Numerous microorganisms and arthropods are capable of transforming plant tissues into galls. The galling habit is especially common among insects with more than 13,000 known gall-forming species from several orders [1]. Gall induction has evolved convergently among and within various insect lineages and the multiple, independent origins of gall-formation indicate that this phenomenon is highly adaptive. Although the molecular mechanisms of gall induction remain to be unveiled, numerous ecological studies and phylogenetic analyses strongly suggest that the insects are in control of the gall traits, which they exploit for their own benefit [1-6]. Galls are thus considered as an extended phenotype of the inducer’s genes [7].

Serving as “incubators” that promote the development of the insects within, galls may have more than a single adaptive function [8,9]. The proposed advantages of the galling habit fall into three main categories [2,6]: (1) Microclimatic stabilization: galls protect the insects from unfavourable abiotic conditions such as high temperature and low humidity (2) Nutrition: gall tissue provides an abundance of high quality nutrients and (3) Defence: morphology and chemistry of the gall tissue protect the inducing insect from various natural enemies, including predators, parasitoids, pathogens and other herbivores. This notion has been termed the Enemy hypothesis [2].

Improved nutrition and defence is achieved by the insect’s ability to considerably manipulate its host plant’s morphology and physiology [10]. Primary and secondary plant compounds are not randomly distributed in the galled tissue as the inner layers on which the insects feed are enhanced sinks for photosynthates and may have low concentrations of secondary compounds, thus providing better nutrition for the gall insect [8,11-13]. The outer, non-nutritive gall layers, on the other hand, may contain increased amounts of potentially defensive chemicals that could deter antagonists [8,14]. Phylogenetic evidence exists that supports the Enemy hypothesis, in particular with regard to gall morphology [15]. However, the adaptive value of gall chemicals remains uncertain [16] and we are unaware of any study that has experimentally confirmed the role of gall secondary metabolites in reducing mortality by natural enemies, which includes vertebrate herbivores although not explicitly mentioned.

Volatile organic compounds such as terpenoids are emitted by many plant species and mediate a wide array of interactions. These volatiles are generally released in response to insect attack and can be exploited as signals by natural enemies of herbivorous arthropods. Induced volatile emission is therefore regarded as an indirect plant defence mechanism [17,18] although the net benefit for the plant still needs to be shown [19]. In contrast to other herbivorous feeding guilds, only few studies have examined how gall-inducers affect the emission of volatiles [20]. However, these have provided interesting insights, suggesting that in some cases gall-inducing insects can take control over the plant’s defence. Like in many non-galling herbivores, plant volatiles may serve as host location cues for parasitoids of the gall insect *Antistrophus rufus*[21]. Interestingly, this gall wasp is also known to alter the ratios of monoterpenes in its host plant which then serve as a sex pheromone [22]. In another case, gelechiid moths were found to suppress the host plant’s ability to produce volatiles which may help these gall insects to avoid predation or parasitism [23].

**Figure 1:**
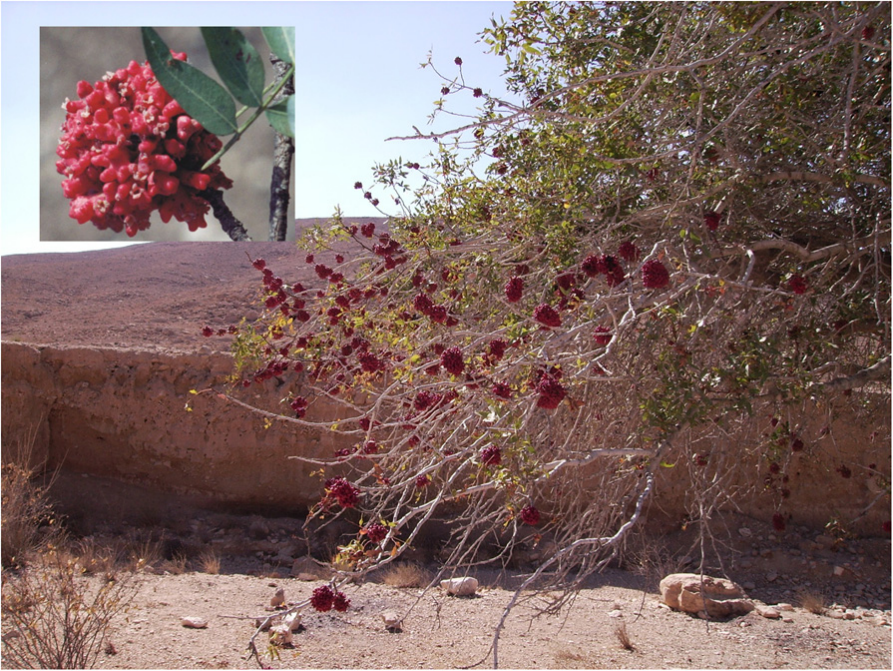
**Cauliflower-shaped galls of *S. wertheimae *on a *P. atlantica *tree.** The galls reach approximately the size of a tennis ball.

In this study we have focused on the role of volatile terpenes in the conspicuous cauliflower-shaped galls that are induced by the aphid *Slavum wertheimae* (Pemphigidae) on the lateral buds of *Pistacia atlantica* (Anacardiaceae) trees (Figure 1) [24,25]. Inside this structure, the fundatrix and her offspring reproduce parthenogenetically and feed on the phloem sap until autumn when galls turn red. By then, the gall may contain thousands of aphids that eventually disperse. Unlike many other gall insects, but similar to North American species in the related genus *Pemphigus*, *S. wertheimae* are not known to be attacked by parasitoids [2,5]. However, anecdotal reports of predation by bulbuls and dipteran and lepidopteran larvae exist [5,26]. Members of the genus *Pistacia* are widespread in Central Asia and the Middle East and serve as obligate hosts for several specialized gall-forming aphid species (Pemphigidae) [27,28]. The galls of these species show several morphological and chemical traits with postulated defensive functions [29-33] that may contribute to protection from natural enemies. One of the risks these galls face is inadvertent predation by various mammalian herbivores such as cattle [34], mountain gazelles [35], camels [36] or goats [37] that browse the leaves of *Pistacia* trees and can also reach galls on large parts of the tree. To further assess the functions of plant volatiles in tritrophic interactions with galls, we explored the following questions: (1) Does gall formation by *S. wertheimae* lead to enhanced concentrations of volatile compounds in the gall? (2) Does increased storage and emission of volatile compounds protect the gall and the insects inside from damage? Individuals of *Capra hircus hircus* (Damascus goat) were used to explore the defensive role of gall volatiles. This species was chosen as a model for a common, non-selective, intensive browser of many plant species including *P. atlantica*. The goat was domesticated from its wild ancestor *C. hircus aegagrus* in the Fertile Crescent region about 10,000 years ago [38] and became an integral and dominant component of local habitats.

Our results demonstrate a new function for plant volatiles by showing that *S. wertheimae* galls are chemically well defended against herbivorous mammals. We speculate that this trait could be adaptive.

## Results

### Total tannin concentrations

**Figure 2:**
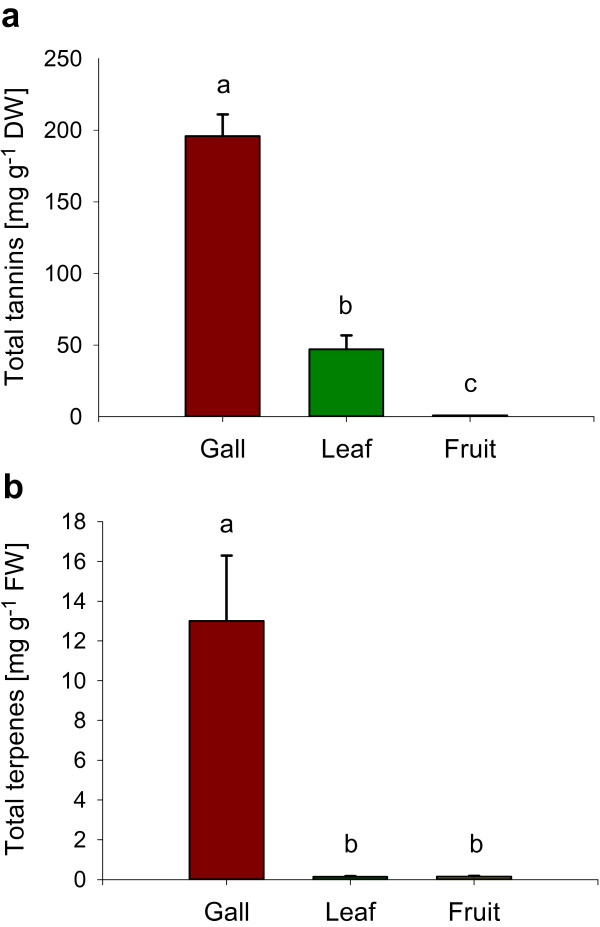
**Secondary metabolite concentrations in galls of *S. wertheimae *and leaves and fruits of *P. atlantica. *(a)** Total tannins (Kruskal-Wallis ANOVA, Chi = 12.000, *n* = 6, *P* = 0.002). **(b)** Total terpenes (Kruskal-Wallis ANOVA, Chi = 14.216, *n* = 6–8, *P* = 0.001). Bars show means ± standard errors. Different letters above bars indicate significant differences.

To obtain a more comprehensive picture of the defence chemistry of *S. wertheimae,* we assessed levels of non-volatile tannins in galls and compared these with levels in leaves and fruits of *P. atlantica*. Aphid galls contained nearly four times higher amounts of total tannins than leaves (Figure 2a). No tannins were detected in any of the *P. atlantica* fruit samples.

### Stored terpene concentrations

**Figure 3:**
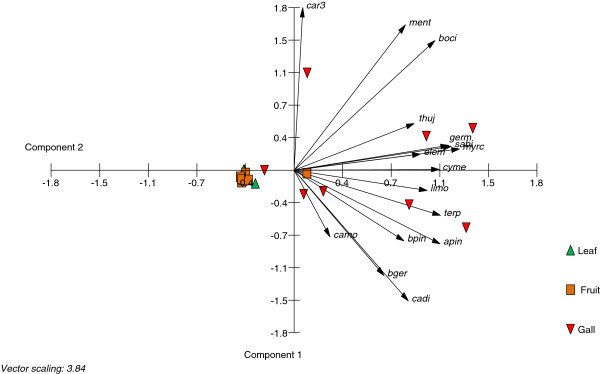
**Biplot of principal component 1 and 2 from PCA on terpenes stored in tissues of galls, leaves and fruits.** Percentage of eigenvalues: component 1 = 45.040, component 2 = 13.555. Compound identities are listed in Table 1.

Comparative measurements of extracted terpenes from gall, leaf and fruit tissues were carried out. The analyses revealed large differences in numbers and quantities of detected compounds (Figure 2b, Table 1). Twenty mono- and 13 sesquiterpenes were identified from *S. wertheimae* galls with the three monoterpenes α-pinene, sabinene, and limonene accounting for 67% of the total compounds. These monoterpenes were also the main components in the tissues of leaves and fruits. Sesquiterpenes made up for 11% of total terpenes in galls, 36% in leaves and only 2% in fruits. In total, the concentration of terpenes was almost two magnitudes higher in galls than in leaves or in fruits (Figure 2b). A principal component analysis clearly separated galls from leaves and fruits. The terpenes strongly correlated with the galls, yet substance profiles of individual galls were variable (Figure 3).

**Table 1:** **Stored terpenes in *S. wertheimae *galls and in leaves and fruits of *P. atlantica***

			**Gall**		**Leaf**		**Fruit**	
**Compound**	**Code**		**μg g**^**-1 **^**FW**	**% total**	**μg g**^**-1 **^**FW**	**% total**	**μg g**^**-1 **^**FW**	**% total**
α-Thujene	thuj	M	34.1 ± 18.1	0.41	n.d.	0	3.5 ± 0.1	3.62
α-Pinene*	apin	M	3283.4 ± 1040.2	24.35	11.5 ± 2.3	11.27	24.4 ± 3.0	24.94
Camphene*	camp	M	37.6 ±13.1	0.26	2.4 ± 0.8	2.32	1.1 ± 0.3	1.15
Sabinene*	sabi	M	4119.1 ± 795.1	28.59	19.4 ± 7.2	19.05	24.4 ± 0.3	24.94
β-Pinene*	bpin	M	783.1 ± 449.0	5.15	3.9 ± 1.1	3.87	11.4 ± 2.0	11.61
β-Myrcene*	myrc	M	170.4 ± 40.7	1.54	n.d.	0	1.2 ± 0.3	1.24
α-Phellandrene*	aphe	M	305.1 ± 137.5	1.91	n.d.	0	n.d.	0
3-Carene*	3car	M	487.3 ± 476.3	10.33	n.d.	0	n.d.	0
α-Terpinene	ater	M	17.2 ± 7.6	0.15	n.d.	0	n.d.	0
p-Cymene*	cyme	M	7.1 ± 2.7	0.05	n.d.	0	n.d.	0
Limonene*	limo	M	2148.1 ± 681.0	14.35	25.8 ± 5.2	25.40	25.8 ± 0.03	26.37
(*E*)-β-Ocimene*	boci	M	4.3 ± 1.1	0.06	n.d.	0	n.d.	0
γ-Terpinene*	gter	M	29.3 ± 14.0	0.25	n.d.	0	1.8 ± 0.01	1.89
(*E*)-Sabinene hydrate*	tshy	M	15.5 ± 5.3	0.18	n.d.	0	n.d.	0
p-Mentha-2,4(8)-diene	ment	M	95.0 ± 40.7	1.10	n.d.	0	2.3 ± 0.1	2.35
(*Z*)-Sabinene hydrate*	cshy	M	14.7 ± 5.0	0.18	n.d.	0	n.d.	0
Camphor*	camo	M	1.9 ± 0.5	0.04	2.4 ± 0.8	2.40	n.d.	0
Terpinene-4-ol*	terp	M	37.8 ± 16.6	0.37	n.d.	0	n.d.	0
α-Terpinolene	ater	M	31.7 ± 10.3	0.20	n.d.	0	n.d.	0
Bornyl acetate*	boac	M	18.4 ± 6.5	0.14	n.d.	0	n.d.	0
δ-Elemene	dele	S	4.2 ± 1.9	0.04	n.d.	0	n.d.	0
α-Cubebene*	acub	S	10.2 ± 3.6	0.09	n.d.	0	n.d.	0
α-Copaene*	acop	S	3.3 ± 1.1	0.02	n.d.	0	n.d.	0
β-Elemene	bele	S	8.0 ± 3.7	0.09	n.d.	0	n.d.	0
(*E*)-Caryophyllene*	tcar	S	80.8 ± 50.1	0.58	n.d.	0	n.d.	0
γ-Elemene	gele	S	5.6 ± 2.6	0.06	n.d.	0	n.d.	0
α-Humulene*	ahum	S	4.2 ± 1.7	0.02	n.d.	0	n.d.	0
Germacrene D*	germ	S	182.9 ±60.1	1.92	34.5 ± 8.7	33.9	1.9 ± 0.5	1.91
Bicyclogermacrene	bger	S	872.7 ± 630.3	5.58	n.d.	0	n.d.	0
δ-Cadinene	dcad	S	4.0 ± 1.9	0.02	n.d.	0	n.d.	0
Cadina-1,4-diene	cadi	S	3.0 ± 1.3	0.02	n.d.	0	n.d.	0
Elemol	elem	S	81.4 ± 41.6	0.61	n.d.	0	n.d.	0
Germacrene B	gerb	S	36.7 ± 16.8	0.37	1.9 ± 0.8	1.90	n.d.	0

*Compound identified by comparison with authenticated standard. Tentative identification of other compounds by comparison of retention indices and mass spectra with Wiley 275 and Massfinder/Terpenoids library databanks. *M* Monoterpene or derivative, *S* Sesquiterpene or derivative, *n.d.* not detected. Mean values ± standard errors are given.

### Volatile emission

**Figure 4:**
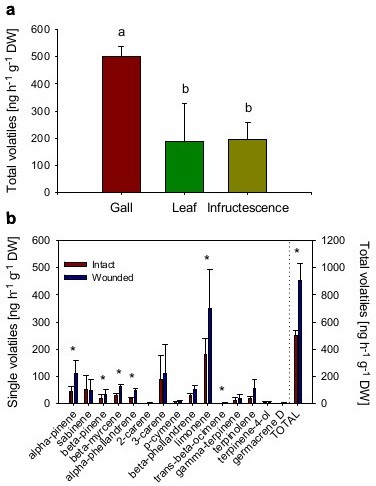
**Volatile emission by *S. wertheimae *galls and *P. atlantica *leaves and infructescences*. *(a)** Emission of total volatiles (ANOVA followed by LSD: *F* = 4.139, *n* = 6, *P* = 0.046). Different letters indicate significant differences **(b)** Comparison of single and total volatile emission in intact and mechanically wounded *S. wertheimae* galls. (single volatiles: Wilcoxon Matched Pairs Tests, *P* < 0.05; total emission: Wilcoxon Matched Pairs test, Z = −2.023, *n* = 6, *P* = 0.043). Bars show means ± standard errors.

**Figure 5:**
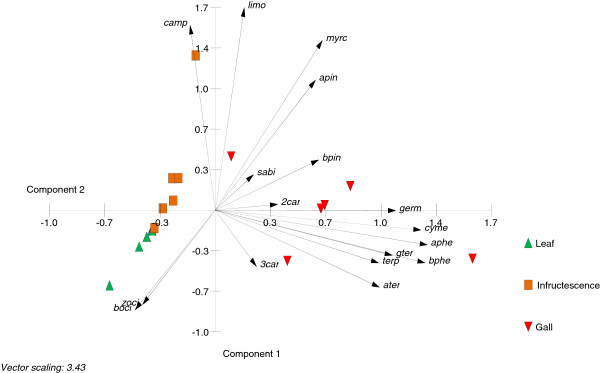
**Biplot of principal component 1 and 2 from PCA on volatile compounds emitted by galls, leaves and infructescences.** Percentage of eigenvalues: component 1 = 36.375, component 2 = 18.623. Compound identities are listed in Table 2.

The amount of volatile terpenes emitted by intact galls of *S. wertheimae* was found to be significantly higher than the emission by leaves or infructescences (Figure 4a; Table 2). Sixteen monoterpenes and the sesquiterpene germacrene D were identified from the headspaces of galls with limonene, 3-carene, sabinene and α-pinene as dominant compounds. In leaves and infructescences only five and seven monoterpenes, respectively, were above the detection limit. Volatile release rates were approximately the same in leaves and infructescences of *P. atlantica.*Both plant parts, however, emitted ca. 2.5 times less terpenes than galls (Figure 4a). Limonene, sabinene and α-pinene were the main compounds in the infructescences, as well. Principal component analysis separated galls from leaves and infructescences. Most measured terpenes correlated with the galls, however, camphene (camp) was associated with infructenscences. The emission of (*E*)-β-ocimene (boci) and (*Z*)-ocimene (zoci) was characteristic for leaves (Figure 5, Table 2).

**Table 2:** **Volatile compounds emitted by *S. wertheimae *galls and by leaves and infructescences of *P. atlantica***

			**Gall**		**Leaf**		**Infructescence**	
**Compound**	**Code**		**ng h**^**-1**^ **g**^**-1 **^**DW**	**% total**	**ng h**^**-1**^ **g**^**-1 **^**DW**	**% total**	**ng h**^**-1**^ **g**^**-1 **^**DW**	**% total**
α-Pinene*	apin	M	43.6 ± 17.5	8.7	2.5 ± 0.8	1.4	16.3 ± 4.7	8.3
Camphene*	camp	M	n.d.	0	n.d.	0	0.5 ± 0.2	0.3
Sabinene*	sabi	M	51.1 ± 50.0	10.2	n.d.	0	69.7 ± 31.8	35.7
β-Pinene*	bpin	M	18.6 ± 13.0	3.7	1.1 ± 0.8	0.6	9.5 ± 2.5	4.9
β-Myrcene*	myrc	M	30.5 ± 4.8	6.1	n.d.	0	11.6 ± 5.8	5.9
α-Phellandrene*	aphe	M	16.7 ± 3.8	3.3	n.d.	0	n.d.	0
2-Carene*	2car	M	0.8 ± 0.8	0.2	n.d.	0	n.d.	0
3-Carene*	3car	M	88.7 ± 88.6	17.8	n.d.	0	n.d.	0
p-Cymene*	cyme	M	5.7 ± 0.8	1.1	n.d.	0	n.d.	0
β-Phellandrene	bphe	M	27.6 ± 9.8	5.5	n.d.	0	n.d.	0
Limonene*	limo	M	181.0 ± 57.2	36.2	9.8 ± 6.6	5.2	87.3 ± 24.9	44.7
(*Z*)-Ocimene	zoci	M	n.d.	0	65.6 ± 48.0	34.6	n.d.	0
(*E*)-β-Ocimene*	boci	M	1.0 ± 0.4	0.2	110.5 ± 80.1	58.3	0.5 ± 0.6	0.3
γ-Terpinene*	gter	M	12.3 ± 8.1	2.5	n.d.	0	n.d.	0
Terpinene-4-ol*	terp	M	3.9 ± 3.3	3.5	n.d.	0	n.d.	0
α-Terpinolene	ater	M	17.3 ± 8.8	0.8	n.d.	0	n.d.	0
Germacrene D*	germ	S	0.7 ± 0.3	0.1	n.d.	0	n.d.	0

*Compound identified by comparison with authenticated standard. Tentative identification of other compounds by comparison of retention indices and mass spectra with Wiley 275 and Massfinder/Terpenoids library databanks. *M* Monoterpene or derivative, *S* Sesquiterpene or derivative, *n.d.* not detected. Mean values ± standard errors are given.

Few needle pricks significantly increased the total amount of emitted gall terpenes by 80%. Qualitative changes in the compound blend were observed as not all monoterpenes were emitted in larger amounts following mechanical damage (Figure 4b). Significantly increased emission rates were found in α-pinene, β-pinene, β-myrcene, β-phellandrene, limonene and (*E*)-β-ocimene.

### Goat behaviour

Individual goats that were offered a single branch of *P. atlantica* completely consumed all of the leaves but none of the galls. Two out of ten goats briefly tasted a single gall but were quick to let it drop. Otherwise, galls were assessed without contact.

**Figure 6:**
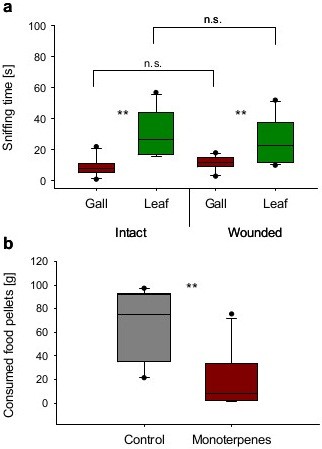
**Behavioural responses of *C. hircus hircus. *(a)***S. wertheimae* galls and *P. atlantica* leaves were offered either intact or slightly wounded. Time spent assessing each odour by olfaction was recorded. Boxes depict medians and quartiles, whiskers and dots show extreme values and outliers, respectively. Asterisks indicate significant differences at *P* < 0.05 (Wilcoxon Matched Pairs tests, *n* = 10; intact: Z = −2.803, *P* = 0.005; wounded: Z = −2.601, *P* = 0.009), wounding had no significant effect (Bonferroni-corrected α = 0.025) on the duration of sniffing (galls: Mann–Whitney U test, U = 24.50, *P* = 0.053; leaves: U = 39.50, *P* = 0.427). **(b)** Consumption of food pellets treated with *α*-pinene, sabinene, and limonene. (Student’s *t*-test for pairs, *t* = 3.475, *P* = 0.007).

In the dual-choice olfaction tests, goats significantly preferred the scent of *P. atlantica* leaves (Figure 6a). On average, animals spent at least twice as much time assessing leaf samples than *S. wertheimae* galls. This was the case for intact as well as wounded leaves and galls. However, wounding had no significant effect on the duration of sniffing.

Goats that were given a choice between food pellets treated with the three main gall monoterpenes and untreated pellets significantly consumed more than twice as much of the latter (Figure 6b).

## Discussion

The galls induced by *S. wertheimae* on wild pistachio trees contained and emitted large amounts of volatile terpenes that deterred goats from feeding. This presents a new function for plant volatiles where the insect uses a host defence trait for its own protection. Our findings also support the frequently debated, but to our knowledge, unproven hypothesis that gall secondary metabolites can confer defence against natural enemies.

Trees and shrubs in the genus *Pistacia* are known to produce both tannins and terpene-containing resins and therefore have a long history in traditional folk medicine [39,40]. Our data confirmed the existence of high levels of tannins in the leaves of *P. atlantica* and furthermore show that their concentrations in aphid galls were four-fold higher. Increased accumulation of tannins and other phenolics have previously been reported from a range of other gall insects [14,16].

Likewise, high amounts of mono- and sesquiterpenes were found in gall tissues of which a subset was measured in the leaves and fruits of *P. atlantica*. The dominant components in all assessed structures and the headspaces of galls were the three monoterpenes α-pinene, sabinene and limonene. Terpene concentrations measured in the tissues of galls, leaves and fruits/infructescences differed considerably more than terpene levels in the headspaces of these structures. We conclude that this was due to employing different sampling methods (destructive solvent extraction from tissue versus non-destructive volatile trapping from headspace) that do not allow for direct comparison of stored and emitted terpenes. In *Pistacia* spp., terpenes are mainly present in the resin ducts of the plant and destructive gall tissue sampling will therefore yield large quantities. However, it was unequivocal that intact galls constitutively emitted larger amounts of mono- and sesquiterpenes than the surrounding plant tissues and thus should be chemically more apparent.

Terpenes are known to have strong biological activities and they are involved in plant defences [41,42]. The analysed gall chemicals influenced the feeding behaviour of the Damascus goats: animals that were offered leaves and galls attached to a twig of *P. atlantica* completely rejected the galls but readily consumed every leaf. In this food selection process, initial olfactory assessment of the potential food items had played an essential role because volatiles emanating from galls signalled that galls were unpalatable. Increasing the amount of released volatiles by 80% as a result of minor mechanical damage, however, did not further reduce the animals’ interest. We speculate that stronger emission is necessary to see dose-dependent effects. Our data further suggest that the presence of the three main terpenes could readily explain the avoidance response of the mammals. Food pellets treated with these volatiles (67% of total terpenes) in concentrations found inside gall tissues significantly reduced their palatability (Figure 6b). The observed rejection after olfactory and gustatory evaluation was not as strong as in intact galls because all food was initially sampled and therefore it can be conceived that other terpenes and/or the high tannin content of galls may have contributed to their low attractiveness. Whether goats had an innate aversion against galls or whether their choices were affected by negative experience in the past remains unknown. However, short-term learning can be excluded as each goat was used only once per experiment. The defensive role of tannins awaits further exploration and it is possible that the high tannin levels found in galls have also contributed to their unpalatability. However, goats are known to be more tolerant to tannin-rich diets than other mammalian herbivores as, for example, sheep. It has been suggested that this is because goat saliva contains increased amounts of inducible proteins that can precipitate tannins [37,43].

To date, numerous studies have generated a detailed picture of the multiple roles that volatile plant components like terpenes play in the interaction with insects or even birds [44,45]. These volatiles are an important part of the plant’s defensive arsenal and act directly by reducing herbivore damage or indirectly by attracting the herbivore’s natural enemies [46,47]. Surprisingly, the relationship between plant volatiles and gall insects, which are highly evolved herbivores with sophisticated biology and physiology [48], has been explored rather rudimentarily. Limited knowledge suggests that the advantage in the co-evolutionary arms race can be on either side, the plant’s or the insect’s, depending on the specific interaction. While it has been shown in at least one case that plants emit volatiles in response to a galling herbivore that can help parasitoids to locate their host [21], it has also been demonstrated that gall insects are capable of suppressing the treacherous response [23]. Adding to this, our findings suggest a different strategy where volatile emission is largely increased in the gall and thus repels mammal herbivores that can inadvertently destroy a whole aphid colony. It can be speculated that the emission of volatiles from the galls of *S. wertheimae* may promote easy detection by predators but such a trade-off has yet to be investigated.

Given that galls on the top of trees face a low risk of browsing, one could speculate that these may contain fewer defence compounds. However, gall chemicals may also protect against frugivorous and insectivorous birds that can prey on the aphids [26]. While this needs further clarification, we have found that bulbuls were deterred by gall compounds when mixed into artificial diet (Inbar, unpublished data).

Because galls are sessile, long lasting, and often conspicuous, there has been strong selection for gall defences against a variety of natural enemies including pathogens, parasitoids, predators and also herbivores. Inquilines that feed on internal gall tissues can play an important role as they have been shown to directly or indirectly kill the gall-forming insect [49]. Protection can be achieved by defensive behaviors of the gall inducer [50,51], physical gall traits (e.g. hardness, thickness, structural complexities) or secondary metabolites [6,14]. The notion that gall chemicals are an adaptive defence is supported, for instance, by the positive correlation between tannin content in oak leaves and the density of cynipid galls on these trees [52]. Adding to this, tannin content correlated negatively with mortality due to fungal infection in the cynipid wasp *Dryocosmus dubiosus*[53].

In our study we show that insect galls create a distinct headspace which is different from the surrounding plant tissues and hence push the borders of their extended phenotype. Volatiles emanating from galls may serve as a first line in the insect’s defence and constitute an honest signal as they can warn potential predators or herbivores before damaging the gall which would result in exposure to high levels of stored chemicals. Recently, the adaptive nature of gall conspicuousness has been addressed [27] and, among other hypotheses [54], it has been suggested that some galls may be aposematic. Galls heavily protected by defensive metabolites could advertise this feature by using warning signals. The strong release of volatiles in conjunction with the gall’s red colouration supports the aposematic gall hypothesis as both increase gall conspicuity. The combination of multimodal warning displays may stimulate several sensory cascades that can promote learning and memory efficiencies in potential enemies [55].

## Conclusions

Unlike in free-living aphids that actively escape goat predation [56], the protection of the gall-inducing aphids studied here depends on plant traits. We unequivocally demonstrated that galls accumulate large amounts of defensive secondary metabolites that protect the aphid colony within from a generalist herbivore. Therefore, it seems plausible that this trait is adaptive. However, further evidence is necessary to support the idea that browsers exert enough selection pressure to have this influence on the gall’s phenotype. Alternative explanations of why *S. wertheimae* galls contain considerably higher concentrations of well-known defence compounds than their host plants are possible. For instance, secondary metabolite accumulation could be an unavoidable physiological side-effect of gall formation that also happens to be defensive. So far, the molecular and biochemical mechanisms that lead to gall formation are not understood in enough detail to either refute or support this notion. Comparing gall defences that have been subjected to different selection pressures from natural enemies over time may answer the question of adaptiveness. Recent evidence suggests that herbivore-driven evolution of plant defences may need only few generations [57].

In *S. wertheimae* volatile emission serves as an antagonistic signal and may be regarded as the outer boundary of the gall inducer’s extended defensive phenotype. Our findings thus add a new direction to the growing body of evidence that illustrate the multiple ecological functions accomplished by plant volatiles.

## Methods

### Study system

*P. atlantica* are deciduous shrubs or trees (3–15 m high) with a wide crown [58]. Sampling of plant and gall material and *in situ* volatile collections were carried out on mature *P. atlantica* trees in a randomized block design with each tree as one block from which we sampled one neighbouring gall, leaf and fruit bunch from September until October 2008. The trees were heavily-galled throughout the canopy and were growing in the area of Tiv’on, Lower Galilee, Israel (32° 42′ 40″ N and 35° 06′ 35″ E). Sampled galls, leaves and fruits were comparable in their developmental stages and located on the branches (< 2 m height) that were within the reach of mammalian browsers. The galls were about five months old which corresponds to the period when the aphid colony inside reaches its developmental peak. Experiments with adult goats were performed in autumn 2011. The animals were part of a small flock used for herbivore feeding studies in the Mediterranean woodlands at Ramat Hanadiv, Israel. The animals foraged regularly on natural vegetation in the forest and received supplemental feed.

### Analyses of tannins

Total tannins (condensed and hydrolysable) were determined by using the radial diffusion assay [59]. Leaves, fruits and galls (*n* = 6 trees) were dried at 80°C for 5 days. The plant material was weighed (100 mg) and homogenized in a mixer mill at 30 Hz for 1 min. Peripheral tissues were extracted with 500 μl aqueous ethanol (50%, v/v) for 1 h at room temperature. Then, extracts were centrifuged at 13,000 rpm for 2 min, the supernatant was transferred into 1.5 ml vials and frozen at −20°C. The next day, an agarose gel (1%, w/v) was prepared containing 50 mM acetic acid, 60 μM ascorbic acid and 0.1% (w/v) bovine serum albumin (BSA). For quantification, eight concentrations of tannic acid solutions were prepared to obtain a calibration curve. Holes of 2 mm in diameter were punched out from the gel using a cork borer and 24 μl of plant extract or tannic acid solution was transferred into the holes. Each extract was filled into two holes to allow for duplicate measurements. Following an incubation period of 42 h at 30°C, tannins had diffused into the agar and created a radial zone by precipitating the BSA. Radial zone diameters were measured and tannin concentrations were calculated from the calibration curve.

### Extraction of terpenes

Frozen leaf, fruit and gall tissues were extracted and assessed for their terpene contents (*n* = 6–8 trees). About 100 mg of plant material was transferred to a 4 ml glass vial containing 1 ml chloroform and 400 ng nonyl acetate as an internal standard. All samples were vortexed for 1 min and transferred to a new glass vial using a glass syringe. Charcoal was added to bind chlorophyll and other contaminants and extracts were vortexed a second time. The suspension was then transferred to a Pasteur pipette filled with Na_2_SO_4_ to a height of 1.5 cm. An aliquot (100 μl) of the filtrate was transferred to a 1.5 ml glass vial and spiked with a second internal standard (400 ng octadecane). Subsequently, terpene contents were analysed and quantified by GC-MS as described below.

### Field-based volatile collections

In a first round of sampling, headspace volatiles of intact galls, leaves and infructescences (drupes with stalks) were collected from *P. atlantica* (*n* = 6 trees) in a replicated field experiment. In a second round, we sampled volatiles from the same galls and leaves but pricked the plant parts seven times with a needle immediately before collection commenced. Sampling was carried out two to three days later but during the same daytime as the first collection. On each tree, a single gall, a pinnately compound leaf and an infructescence were enclosed with plasticizer-free PET foil (Toppits Brat-Schlauch, Germany). Each bag was connected to a portable battery-operated air pump (PAS-500, Spectrex, CA, USA) by a short PTFE tube (L: 30 mm, ID: 4 mm). To avoid condensation, bags were shaded by attaching a sheet of white paper to a nearby twig. As a control, a fourth pump was attached to a twig without using a bag to sample the air in the canopy. Air from the bag was pulled through a volatile collector trap containing 30 mg Super-Q (Analytical Research Systems, FL, USA) at a rate of 200 ml min^-1^. After a 6 h collection period, volatiles were eluted with 150 μl methylene chloride and two internal standards (*n*-octane and nonyl acetate, each 200 ng in 10 ml methylene chloride) were added. Samples were stored at −80°C until analysed as described below.

### Analysis of terpenes

Aliquots (3 μl) of the samples were analysed by gas chromatography–mass spectrometry (GC: HP 6890 N, MSD: Agilent 5975) equipped with a split/splitless injector and a HP-1 ms column (30 m × 0.25 mm internal diameter, 0.25 μm film thickness). Samples were injected in pulsed splitless mode. Inlet temperature was 230°C. The oven was held at 35°C for 3 min and then programmed at 8°C min^-1^ to 230°C, where it was maintained for 9.5 min. Helium (1.5 ml min^-1^) was used as carrier gas. Compound identities were confirmed by comparison with mass spectra and retention indices of the Wiley 275 and Massfinder 3/Terpenoids libraries as well as co-injection of standards (Sigma-Aldrich, Germany and RC Treatt Ltd., Suffolk, UK). Quantification of compounds was based on comparison with the internal standards.

### Goat bioassays

Choice experiments with individually caged Damascus goats (*C. hircus hircus*) were conducted to assess their olfactory and gustatory responses to galls of *S. wertheimae*. Each goat was used only once throughout the three choice tests. All plant material was harvested randomly from the same *P. atlantica* trees that were sampled for the analyses of chemical compounds.

In the first assay we tested the palatability of aphid galls in comparison to leaves. Each goat (*n* = 10) was offered a single shoot (20–30 cm long) with five to seven leaflets and two attached galls. The shoot was placed on a pedestal at 0.5 m height inside each cage. The number of consumed galls and leaflets was assessed after 5 min.

The second bioassay tested the animals’ olfactory responses to gall and leaf odours. Either 300 ± 10 g of intact leaves or galls were placed inside a wire mesh basket that was confined to the rear side of a plastic tub (38 × 33 × 15 cm). A wooden board covered the tub almost entirely with only a slit (33 × 7 cm) at the opposite end of where the basket was placed remaining open. This allowed the animals (*n* = 10) to smell the test material without seeing it. Both boxes were adjacently placed into the cage of a goat. For all cages the positions of the boxes were randomized. The time each goat spent sniffing at either box was recorded for 7 min.

The experiment was repeated with new goats (*n* = 10) in the same manner but leaves and galls were wounded with seven needle pricks before being placed into the boxes.

A third experiment was conducted to confirm the role of gall terpenes as feeding deterrents. Every goat (*n* = 10) was offered two plastic cups on the cage floor, each containing either 100 g of treated or untreated feed pellets (Amir Dagan Feed Mill, Kiryat Haim, Israel). The pellets consisted of wheat, corn, sunflower meal and soy hulls and were routinely given to the flock as protein supplement. The position of each cup was randomized for each goat and trial. Treatment consisted of mixing food pellets with pure terpenes in the same concentrations as found in the gall tissue α-pinene (3.8 μl/g), sabinene (4.9 μl/g) and limonene (2.5 μl/g) (racemic mixtures; Sigma-Aldrich, Israel). Each goat was allowed to feed for 3 min. The remaining pellets in the cup were weighed at the end of the trials.

### Statistical analyses

Total amounts of tannins and stored terpenes were compared by Kruskal-Wallis ANOVA followed by median tests. Total volatile emission of samples was analysed by one-way ANOVA followed by LSD tests, while Wilcoxon matched pairs tests were used for comparing differences between volatile compounds before and after wounding. Olfactory choices of goats were assessed by Wilcoxon matched pairs tests. Differences in their olfactory responses to intact and wounded materials were compared with Bonferroni-corrected Mann–Whitney U tests. A Student’s *t*-test for pairs was carried out to analyse the amounts of consumed food pellets. SPSS Statistics 20 (IBM Corp.) was used for all analyses.

We further investigated the profiles of stored and headspace terpenes by principal component analysis (PCA) using R 2.15.0 (The R foundation for statistical computing). With stored terpenes, cluster analysis on Pearson’s correlation coefficients was performed between each pair among 33 variables to reduce the number of variables prior to PCA. Fifteen clusters of co-related compounds were generated of which the most abundant compound per cluster entered PCA. In contrast to stored terpenes, the whole data set could be used to analyse headspace compounds.

## Abbreviations

BSA: Bovine serum albumin; GC-MS: Gas chromatography – mass spectrometry; PCA: Principal component analysis; PET: Polyethylene terephthalate; PTFE: Polytetrafluoroethylene.

## Competing interests

The authors declare that they have no competing interests.

## Authors’ contributions

MI and MR contributed in equal parts. They designed the study, wrote the manuscript and carried out volatile collections. DM, MIK and MR analysed secondary metabolites, MI performed behavioural experiments. All authors read and approved the final manuscript.
